# Formative Work to Guide Implementation of an Evidence-Based Breast and Cervical Cancer Prevention and Early Detection Behavioral Health Program in Clinics: Qualitative Study Guided by the Consolidated Framework for Implementation Research

**DOI:** 10.2196/89393

**Published:** 2026-08-13

**Authors:** Marlyn A Allicock, Crystal Costa, Ashley Hedrick McKenzie, Preena Loomba, Roshanda S Chenier, Ross Shegog, Angelita Alaniz, Maria E Fernandez, Lara S Savas

**Affiliations:** 1 The University of Texas Health Science Center at Houston, 2777 N Stemmons Fwy, Dallas, TX, 75207, United States, 1 972-546-2932, 1 214-648-1081; 2 Department of Communication, Clemson University, South Carolina, SC, United States; 3 The University of Texas MD Anderson Cancer Center, Houston, TX, United States; 4 The University of Texas Health Science Center at Houston, 7000 Fannin, Suite 2668Houston, Texas, TX 77030‑5401, United States

**Keywords:** implementation science, consolidated framework for implementation research, breast and cervical cancer screening, lay health workers, latinas, federally qualified health centers, FQHCs

## Abstract

**Background:**

Salud en Mis Manos (SEMM) is a community health worker–delivered evidence-based intervention shown to increase breast and cervical cancer screening in medically underserved Latinas. Despite SEMM’s success in community-based settings, clinic-based implementation is limited.

**Objective:**

This study aimed to identify factors influencing the implementation of SEMM in clinic settings using the Consolidated Framework for Implementation Research (CFIR) to inform and refine implementation strategy development.

**Methods:**

We developed theory-informed (eg, CFIR, Social Cognitive Theory, and Readiness) interview guides to elicit barriers and facilitators of SEMM clinic implementation. We conducted 12 semistructured interviews with clinic leaders, outreach coordinators, and community health workers from 4 Texas health centers serving low-income Latinas. Transcribed interviews were coded and analyzed using qualitative thematic analysis and CFIR.

**Results:**

Barriers and facilitators fell within 17 constructs of the 5 CFIR domains. Across all interview participants, key facilitators fell within intervention characteristics (eg, SEMM addresses community needs), inner setting (eg, clinic staff buy-in can facilitate SEMM implementation), and outer setting (eg, established partnerships may leverage SEMM uptake) domains. Barriers were inner setting (eg, limited time and clinic staff capacity may limit SEMM implementation) and intervention characteristics (eg, challenges with SEMM training duration and content).

**Conclusions:**

CFIR, a theory-based systematic approach, enabled key stakeholder-identified barriers and facilitators to SEMM clinic implementation. These factors help identify implementation support strategies distinct from prior community-based implementation efforts. Findings (part of the initial steps of the Implementation Mapping protocol for strategy development) refined the design of a multifaceted implementation strategy to support SEMM clinic uptake.

## Introduction

Uninsured and underinsured women, as well as those from traditionally underserved minority groups such as Latinas, are more likely to underuse cervical and breast cancer screening, resulting in later-stage detection and lower survival rates [1,2]. Interventions to increase breast and cervical cancer screening that use approaches recommended by the Guide to Community Preventive Services, such as community health workers (CHWs) delivering one-on-one or group education, have demonstrated effectiveness in increasing screenings [3]. The Salud en Mis Manos (SEMM) program [4,5] uses an evidence-based CHW-delivered model to provide breast and cervical screening education and health coach navigation to low-income Latinas in several health community settings. CHWs are trained to provide Latinas who have unmet breast and cervical screening needs with (1) education, (2) referral to low-cost services, and (3) ongoing health coaching and navigation support to address personal, psychosocial, and health care access barriers. In brief, developed for Mexican American women living in farmworker communities [5,6]. It was adapted for medically underserved Latina adults to include telephone-based health coaching and a navigation component delivered by coach navigators trained to help women overcome structural and personal barriers in order to obtain cancer prevention services. SEMM has three components intended to increase breast and cervical cancer screening: (1) a bilingual CHW-led group education session, based on the original curriculum (using a flipchart and optional video, which became outdated and dropped over time) to address psychosocial determinants of screening, (2) a clinic referral database developed for the new setting, used by CHWs to provide participants with tailored lists (based on residence) of local and affordable clinics where they could access recommended screenings, and (3) a new telephone-based health coaching and navigation protocol delivered by bilingual health coach navigators. The group education is a one-hour-long session using behavior change techniques, such as role models, testimonials, and persuasive communication to convey cancer screening messages targeting psychosocial determinants of screening, and to increase knowledge of screening guidelines [5,6]. The optional health coach navigation assistance includes ongoing phone calls by trained health coaches (lay health workers or CHWs) to provide personalized support and to assist participants in overcoming complex barriers to screening. The adapted version effectively increased screening in the intervention compared with control groups for both mammogram (39.9 vs 20.3%; *P*<.001) and cervical screening (55.8 vs 27.4%; *P*<.001); intent-to-treat analyses were also significant for women in the pap test screening arm [4].

While shown to be effective in community settings [4,7], SEMM has not been widely adopted in clinic settings. The current study aimed to understand barriers and facilitators to adopting and implementing SEMM in clinic settings, an important first step in developing an implementation strategy using Implementation Mapping [8]. Implementation Mapping is a systematic process that facilitates the planning and design of evidence-based interventions for adoption, implementation, and maintenance [9-12]. An assessment of implementation needs and assets is the first step and a critical component of understanding how well organizations are equipped to implement a program [8]. Although there is strong evidence to support the effectiveness of CHWs to improve cancer prevention and control outcomes in both community and clinic settings [4,5,7,13], implementation science research facilitating the transition of cancer screening interventions to clinical environments is limited. Guidance to effectively disseminate and implement evidence-based screening programs into clinic settings is needed. As such, we conducted this qualitative study to inform the needs and assets assessment of community clinics.

## Methods

### Overview

This study was conducted as part of an ongoing Centers for Disease Control and Prevention (CDC)-funded quasi-experimental study (HSC-SPH-20‐1117) aimed at developing and evaluating a multifaceted implementation strategy to support the adoption and implementation of SEMM in clinic settings. This qualitative inquiry included in-depth interviews with key decision-makers and implementers in primary health care settings. We developed semistructured interview guides informed by the Consolidated Framework for Implementation Research (CFIR) [14], Social Cognitive Theory [15], and the Readiness Heuristic, R=MC^2^ (readiness =motivation×innovation specific capacity×general capacity) [16,17]. Social Cognitive Theory informed exploration of individual-level determinants among clinic staff (eg, self-efficacy to implement a new program such as SEMM), while the Readiness Heuristic helped to identify organizational-level readiness constructs (eg, factors that may motivate a clinic to adopt and implement SEMM) [16]. CFIR is a widely used determinants framework in implementation research, well-suited for multilevel interventions, and has been commonly used in health care settings [18]. It is considered a “meta-theory” as it encompasses and blends multiple implementation theories into domains and constructs that are practical and applicable to implementation research. The CFIR outlines 39 constructs organized into five major domains, including (1) intervention characteristics, (2) inner setting, (3) outer setting, (4) individual characteristics, and (5) processes of implementation, from across implementation frameworks that describe the organizational and contextual settings that are thought to influence implementation [14]. CFIR helped inform the development of the interview guide, including the selection of SEMM implementation barriers and facilitators to address in the interview guide. We also used the CFIR as a guiding framework for data coding and analysis.

### Participant Recruitment

A total of 20 federally qualified health centers (FQHCs) or safety-net clinics (hereinafter referred to as clinics) were identified using the UTHealth Houston Center for Health Promotion and Prevention’s community partner contact list. These included primarily FQHCs that had participated in other studies and programs. Study staff sent a recruitment flyer to clinics and scheduled a discovery meeting via telephone. The purpose of the call was to determine the population served by the clinic and whether clinics had CHWs. If eligible, they were then invited to participate in interviews related to the implementation of SEMM. Clinics were eligible to participate in interviews if they were: (1) a clinic with a patient education position, for example, a CHW or lay health worker (LHW) or other staff providing health education or navigation, (2) located in Texas, and (3) primarily serving a Latino/a population. From the list of 20 clinics, 13 clinics were invited to participate based on eligibility. Of these, 9 clinics did not respond. Four clinics were recruited from April to July 2021.

### Setting and Study Population

We conducted semistructured interviews with 3 stakeholders within each of 4 safety-net clinics (n=12 interviews). A sample of 9 (mean of 12‐13) interviews is the established guidance for theme saturation [19]. One clinic had previously partnered with the SEMM team but did not fully implement the program, engaging in some delivery of SEMM education in both community and clinic settings, as part of a prior UTHealth community-based prevention project (CPRIT PP 190061). Thus, responses from this participant may reflect prior experience with SEMM, while other participants could hypothetically reflect on how they imagined the program could be integrated in their clinical setting. Clinics were in Dallas, Houston, and Corpus Christi, TX. Interview participants represented different potential key decision makers and implementers, such as clinic leaders (eg, CEO, Medical Director, n=4), mid-level personnel (eg, Program Director, Clinic Manager, n=4), community health workers (CHWs, n=2), lay health workers (LHWs, n=2). Participants referred to as CHW or LHW are non-professional clinic members acting as an educator, advocate, or liaison to the community on behalf of the clinic. Although these terms may be interchangeable, we use them here as the clinic identifies them.

### Qualitative and Quantitative Data Collection

For all 3 interviewee categories (leadership, mid-level managers, CHW or LHW), we created 3 similar but separate semistructured guides. Questions were asked to understand how each clinic’s current activities related to breast and cervical cancer screening and past approaches to changes in practice aimed at improving screening. They were also asked to describe any factors they believed either facilitated change or created barriers to implementation of SEMM. Before the interviews, participants were sent a PowerPoint presentation that included an overview of the SEMM intervention to prompt their thinking about program fit, needs, and assets for implementation. In scenarios where participants could not review the information ahead of time, the study team presented the PowerPoint at the beginning of the interview. We also collected demographic information using a brief 14-item REDCap hosted at the UTHealth Houston School of Public Health survey once participants completed the interview. The research staff followed up via email with interviewees to thank them for their participation, provide the incentive, and remind them to complete the survey. Four study team members, graduate students who were not involved in the creation or implementation of SEMM, conducted the interviews via Webex. Given that the data collectors had no previous relationship with SEMM, this likely reduced any implicit pressure to speak positively about the program. Data collection teams debriefed after interviews to capture any nuances, identify any issues or clarify how questions were asked, and discuss emerging themes. We stopped data collection when the data collection team felt that saturation was reached.

### Qualitative Data Analysis

Data were thematically analyzed and coded using an iterative-deductive approach using CFIR as the guiding theory [20]. We audio-recorded, transcribed, and deidentified each interview, and imported it into ATLAS.ti (version 9; ATLAS.ti Scientific Software Development GmbH). We used a collaborative, team-based approach to data analysis to enhance analytic rigor, achieving negotiated agreement between multiple researchers for each coding decision. Two researchers (CC and AHM) developed the initial, deductively driven codebook, primarily focusing on the CFIR framework. Other researchers have also used CFIR domains to inform the development of a codebook [18]. The 2 researchers (CC and AHM) jointly coded the first 2 transcripts (one leadership and one mid-level) to refine the codebook definitions and added emergent codes after group discussion with the first author (MA). Next, 2 researchers (CC and AHM) double-coded the remaining 10 transcripts in ATLAS.ti. The researchers used practices for consensus-building during coding [21], such as noting each disagreement between coders and holding several iterative meetings throughout the coding process to resolve coding disagreements. In these meetings, each coder (CC and AHM) described their rationale for their coding decision and collaboratively discussed reasons for disagreement [21]. When coding disagreements involved nuanced themes that were not easily resolved, disagreements were notated and discussed in a collaborative meeting between the coders (CC and AHM) and the first author (MA), who acted as a “tie breaker.” None of the coders were part of the original team that created and implemented SEMM to minimize bias in interpreting the success of SEMM. Findings from our analysis were shared with our study’s 8-member Community Advisory Board (made up of community clinic and CHW agency stakeholders) for feedback and guidance about the findings to inform and guide implementation strategies for the next study phase.

### Ethical Considerations

This study was approved by the UTHealth Houston School of Public Health’s Institutional Review Board (HSC-SPH-20‐1117). We obtained verbal consent from all participants. REDCap survey data and interview transcripts were deidentified and analyzed as stated above. Once individual interviews were completed, leadership and mid-level staff received a one-time $50 gift card, and CHWs received a US $25 gift card for their time.

## Results

### Overview

We conducted 12 interviews; however, we only received survey responses from 11 participants. Participants were able to “check all that apply” for the race category, in which one participant identified as more than one race. Table 1 describes the characteristics of participants and clinics represented in the interview sample. All four clinics were in metropolitan locations and considered urban areas. The barriers and facilitators fell within 17 constructs of the 5 CFIR domains. The most prominent facilitators across all interview participants were in the domains of intervention characteristics and inner and outer settings. Barriers related to the inner setting and intervention characteristics domains. We summarize major findings according to CFIR domains and constructs and provide example quotes as illustrations (Table 2).

**Table 1.** Sociodemographic characteristics of clinic staff.

Participant characteristics (n=11)^a^	Values
Sex, n (%)	
Male	1 (10)
Female	10 (90)
Age (years), mean (SD)	43.9 (8.88)
Ethnicity, n (%)	
Hispanic/Latino	6 (55)
Non-Hispanic/Latino	5 (45)
Race, n (%)	
Black/African American	3 (27)
Other	1 (9)
Non-Hispanic White	8 (73)
Years in role, average (range)	
Leadership	17 (11‐25)
Mid-management	9.6 (2‐16)
Community health worker or lay health worker	8.3 (6‐9)
Clinic characteristics represented, n (%)	
Clinic rural-urban community area code	
Urban	4
Rural	0

a Missing one survey response hence n=11. Race category included “check all that apply” reflecting a percentage higher than 100%.

**Table 2.** Barriers and facilitators to the implementation of Salud en Mis Manos (SEMM).

CFIR^a^ domains and constructs	Barriers	Example quote	Facilitators	Example quote
Relative advantage: stakeholders’ perception of the advantage of implementing the intervention versus an alternative option	Implementation of similar programs may be in conflict with SEMM^b^	And again, sorry to sound repetitive, but it’s all about our plans for the population health team.	Intervention perceived as an additional model of health care Addresses community needs, eg, provides health education, cultivates awareness Intervention appears streamlined	Well, certainly we could be an additional model of health care, a good model of health care, because if we really tried to reach people sooner, while they are either sick now, or getting them early and providing education to the community about a lot of things.
Adaptability: the degree to which an intervention can be adapted, tailored, refined, or reinvented to meet local needs.	Program materials perceived as too structured, need for more flexibility to facilitate tailored approaches	So I would say some flexibility in the application of the program because what works in location A may not work in location B.	Intervention is similar cancer prevention efforts already implemented by clinics Intervention appears “simple to do”	Well, I think implementing it in a clinic like ours is very simple to do. It’s not overly burdensome because we already do it.
Trialability: the ability to test the intervention on a small scale.	—^c^	—	Clinic culture instills willingness to pilot programs such as SEMM	A program like SEMM, I believe in our current clinic culture, I think we’re supportive of new programs. So if it was brought to our clinic, I think that it would be considered.
Complexity: perceived difficulty of implementation, reflected by duration, scope, radicalness, disruptiveness, centrality, and intricacy and number of steps required to implement.	Concerns with risk management of data (eg, data cleaning and entry) SEMM has a long educational component for patients to sit through Number and duration of trainings Repetitive training	Actually, implementing it is really going to come down to, is it going to help the organization? Is it going to become extra work? How well is it going to work with our EMR^d^ system?	—	—
Design quality and packaging: perceived excellence in how the intervention is bundled, presented, and assembled.	Need for SEMM program components to be flexible SEMM has a long educational component for patients to sit through	While we understand it and it’s great education, and we understand that it’s evidence-based, it can be challenging to get a patient to want to sit still... just to listen to education.	SEMM program materials are appealing SEMM program appears streamlined	The program itself looks very streamlined, easy to use.
Needs and resources of those served by the organization: the extent to which patient needs, as well as barriers and facilitators to meet those needs, are accurately known and prioritized by the organization.	Because FQHCs^e^ serve underinsured or uninsured populations, patients often face various barriers to receiving and seeking out health care related to socioeconomic status Virtual and medical appointments missed due to COVID-19–related challenges for patients and staff creating navigation barriers	But right now that’s currently all we have, I’m trying to connect with X organization so that we can offer more services in the X County area for, especially like diagnostics versus patients having to travel all the way to the Houston area, which there’s transportation barriers regarding that one.	CHWs^f^ have roles that evolve to the needs of the community	Oh, they’re kind of promoted. They almost function as social workers in a sense. It’s how their role has evolved to where there’s community needs, and they’re trying to match patients up with needs with other facilities.
Cosmopolitanism: the degree to which an organization is networked with other external organizations.	Not aware of community partners supporting their clinic’s outreach efforts	Can’t think of any.	Existing partnerships with varied organizations (eg, community health centers or local health clinics, major hospitals, mammography vans, grocery stores, Area Health Education Center, local restaurants, community colleges, Lyft and bus systems)	We have partnerships with hospitals and one other organization, I can’t recall right now, that have the mobile mammography unit that comes out.
External policy and incentives: broad constructs that encompass external strategies to spread interventions, including policy and regulations (governmental or other central entity), external mandates, recommendations and guidelines, pay-for-performance, collaboratives, and public or benchmark reporting.	—	—	Clinics already report quality measures to governmental national and/or state agencies eg, Department of State Health Services, Breast and Cervical Cancer Program	We do have [reports] every single year, we do need to report mammogram screenings and cervical cancer screenings, but we don’t necessarily need to report HPV vaccines.
Networks and communication: the nature and quality of webs of social networks and the nature and quality of formal and informal communications within an organization.	—	—	Communication among clinic staff facilitated through provider meetings, one-on-one meetings, and email or phone	Well, just it would be nice... I think whoever’s telling the rest of the staff about the program, like some... or maybe hello emails, "This is the program.”
Implementation climate: the absorptive capacity of an organization to change and the shared receptivity of the involved individuals to an intervention	—	—	Leadership anticipates what they need to do to ensure buy-in from staff Clinic has an “open door policy” for staff members at different levels to bring forward ideas or concerns Previous use of or desire to use tools that demonstrated success (ie, new software, text messages, gifts or incentives for patients) Team-oriented culture makes interventions easier to implement	One of the things that I really want to make sure, is that if, which we believe this particular topic is one of our areas of focus for improving outcomes, the expectation is that we’re all going to implement it, we just need to make sure that the staff members buy into how they want to do that and I would really allow them to come up with the way they want to implement it.
Compatibility: the degree of tangible fit between meaning and values attached to the intervention by involved individuals, how those align with individuals’ own norms, values, and perceived risks and needs, and how the intervention fits with existing workflows and systems.	Changes in software use create issues for routine processes (and likely intervention implementation) Not all CHWs are involved with HPV^g^ vaccination programs Previous mammogram or pap smear programs were too resource/time-intensive to sustain	[When asked if CHWs are involved in HPV vaccination programs] No, I don’t believe so.	SEMM appears similar to previous or future implemented cervical and breast cancer programs Potential easy fit within existing programs for new mothers SEMM aligns with clinics’ core mission to serve and educate Clinic perceives SEMM as a good fit because clinic is the only main care provider “out in this area”	I think it’s definitely a good fit. It’s like a good shoe…If it’s going to help us help our patients, bring it and we’ll do everything we can. And it doesn’t mean that we succeed every time. I wouldn’t dare say that. But I do know that if not us, who? Because we’re going to definitely give it our best effort, and we’re going to have the backing of our board and the leadership of our executive director….We just do it like Nike
Available resources: the level of resources dedicated for implementation and ongoing operations, including money, training, education, physical space, and time.	No CHWs and/or limited staff Limited clinic space Lack of funding or money Scheduling logistics Lack of supplies (vaccines for catch-up population)	If we had more space, which at both sites right now, we do not. I could not implement something like that [SEMM], because if we’re going to bring on more people, we need space to have them have a place to work.	Resources available for new initiatives through external funding or grants Clinics equipped with resources (eg, EHR^h^ systems, mass mailing, text message systems) Availability of quality staff members (eg, coordinators, CHWs) Availability of health promotion materials	So we currently have a state-funded grant called breast and cervical cancer screening, BCCS^i^, that we are able to schedule patients who are uninsured for breast and cervical cancer screening services. We currently also partner with X organization. We have an agreement that we can use our state funding with them.
Access to knowledge and information: ease of access to digestible information and knowledge about the intervention and how to incorporate it into work tasks.	Limited staff, not enough personnel to conduct trainings and disseminate knowledge Some CHWs have limited roles, not involved in ongoing training	I don’t. I don’t do any of that training [speaking of CHW ongoing training].	Some clinics have good infrastructure for training staff and perceive training or education for CHWs as very important Additional education provided by external entities (eg, Pfizer and American Cancer Society) Internal communication introducing SEMM could help with implementation CHWs stressed that training information should be helpful and less redundant	Our approach is to be abreast of industry standards. Collectively, we subscribe to some newsletters, to some entities that provide training webinars, continue education for health
Other personal attributes: other personal traits such as tolerance of ambiguity, intellectual ability, motivation, values, competence, capacity, innovativeness, tenure, and learning style.	—	—	Motivation to have clinic perform well because staff themselves are treated there Staff involved with interventions need to be able to work hard, intrinsic motivation, and have a “heart of service” Leadership with public health backgrounds, rather than regular physicians could facilitate implementation	It’s no doubt the program works, but it’s getting people with the heart of service to carry out the mission
Formally appointed internal implementation leaders: individuals from within the organization who have been formally appointed with responsibility for implementing an intervention as coordinator, project manager, team leader, or other similar role.	—	—	Leadership, medical directors, executive team, CEO, CMO, clinical directors, billing directors, population health team	[the executive director] She’s our fearless leader. She takes us where nobody’s ever thought to take us before…
Champions: individuals dedicated to supporting, marketing, and driving through an implementation, overcoming indifference or resistance that the intervention may provoke in an organization.	—	—	Referred to by clinic leaders as quality coordinator, care coordination staff, CHWs	Our care coordination staff is what we call them, our care coordination staff is working behind the scenes on our quality metrics, cold calling, just hounding patients reminding them of appointments and labs and everything that they need.
Key stakeholders: individuals or organizations with a vested interest in the intervention	Additional buy-in required from appropriate staff for implementation of new programs	I think it’s just going to take talking to the people who are over the CHWs because definitely, it sounds like the vast majority of the work is going to fall on them.	Key leaders often confer with their board or leadership team before implementing something new One participant mentioned that as a leader, she considers the likelihood of staff buy-in before deciding Customer service associates, CHWs, health education departments, and patients also identified as important stakeholders	I’m the one that makes the decisions, but I use the team that I have, our executive team.

a CFIR: Consolidated Framework for Implementation Research.

b SEMM: Salud en Mis Manos.

c Not applicable.

d EMR: electronic medical record.

e FQHC: federally qualified health center.

f CHW: community health worker.

g HPV: human papillomavirus.

h EHR: electronic health record.

i BCCS: breast and cervical cancer screening.

### Intervention Characteristics

The intervention characteristics domain indicates attributes of an intervention that might influence implementation.

SEMM design quality and packaging. Across all clinic levels, participants’ perceptions regarding the design quality and packaging of the intervention were categorized as program facilitators. Clinic leaders and outreach coordinators described SEMM as “structured” and “helpful.” A mid-level staff member who previously implemented SEMM at their clinic mentioned that the program’s educational component was a facilitator for organizations to choose the program.

Relative advantage is defined as the stakeholders’ perception of the advantage of implementing the intervention versus an alternative option [22]. Most participants perceived the use of the SEMM program as an advantage over their current practice. One leader described SEMM as “an additional model of healthcare” that could help “reach people sooner.” Another leader pointed to the benefits for patients, saying “they would receive additional education outside of the physician-patient room.” The CHWs had similar perceptions about the relative advantage of SEMM in providing cancer health education to patients. Additionally, they mentioned that SEMM could also be a beneficial “resource” for clinical staff as it would provide additional cancer-related education. However, a notable barrier was that some clinics already had similar programs or were planning future ones related to cervical cancer, which could conflict with SEMM.

Adaptability is the degree to which an intervention can be adapted, refined, and tailored to meet a clinic’s needs is a factor that can facilitate (or deter) implementation [14,23]. Participants from a clinic that previously implemented SEMM offered insight into the potential for program refinement. For example, while they said that implementing SEMM was “simple” to do, they also noted that they “may not do it in the style that SEMM is presenting it in,” and mentioned tailoring it according to their needs. Participants also mentioned that having “some flexibility in the application of the program” was important to consider, “because what works in location A may not work in location B.” A CHW pointed to the potential redundancy of information provided in SEMM CHW training to educational training they previously had, saying that SEMM may “sometimes have a lot of the information we already know” and suggested more “interactive education” for hands-on learning.

Trialability is the ability to test an intervention on a small scale [23]. Participants viewed the intervention as one that could be carried out as a trial run. One leader believed that SEMM could be tested through their clinic’s existing women’s health campaign. Other mid-level staff commented that because of their clinic’s culture of exploring new ideas, they would be supportive and willing to pilot a new program like SEMM.

Complexity is perceived as the difficulty of implementation, reflected by duration, scope, radicalness, disruptiveness, centrality, intricacy, and number of steps required to implement [23,24]. Participants perceived components of SEMM to be complex because of “the level of” and “amount of” training or education to be provided for patients. In addition, a mid-level staff member, whose clinic previously implemented SEMM, said:

Some of the challenges with implementing the program included its education. While we understand it and its great education, and we understand that it’s evidence-based, it can be challenging to get a patient to want to sit still… just to listen to education.

### Outer Setting

The outer setting domain of CFIR refers to the range of factors involving entities external to the organization that could influence intervention implementation.

Cosmopolitanism, or “the degree to which an organization is networked with other external organizations,” [14] was the most frequently discussed element of clinics’ outer setting. Overwhelmingly, participants at all levels described their clinics as deeply embedded in a network of partnerships; only one participant was unaware of community partners. Clinic partners were diverse, including other clinics and hospitals, local grocery stores and restaurants, community-based organizations, community colleges, bus systems, and ride-sharing apps like Lyft. Participants suggested that clinics relied on those partners for other health initiatives and could leverage these diverse partnerships to serve the needs of their patients for other health promotion programs.

Needs and resources of those served by the organization refer to the extent to which patient needs, as well as barriers and facilitators to meet those needs, are accurately known and prioritized by the organization [14]. The clinics’ perception about the needs and resources of those served by the organization also constitutes an important characteristic of clinics’ outer settings that can potentially facilitate the uptake of an intervention. Primarily, clinics serve underinsured or uninsured populations; their patients often face various barriers to seeking out and receiving health care due to factors related to their socioeconomic status. Participants pointed out that CHWs are critical assets for educating and providing patient navigation to promote the health and well-being of patients. CHWs are described as having a deep understanding of patients’ needs and challenges and having the expertise to help address those needs. CHWs were seen as a way to ensure that the clinic was not only considerate of the needs and resources of the community but also intervening in ways to address some of the issues. Describing the CHWs at his or her clinic, one leadership-level participant said:

They almost function as social workers in a sense. It’s how their role has evolved.where there’s community needs, and they’re trying to match patients up with needs with other facilities.

External policy and incentives refer to Broad constructs that encompass external strategies to spread interventions, including policy and regulations (governmental or other central entity), external mandates, recommendations and guidelines, pay-for-performance, collaboratives, and public or benchmark reporting [25]. External policies and incentives also served as a facilitator to the potential implementation of SEMM. Specifically, leadership and mid-level clinic employees spoke about reports on quality measures they were required to send to government organizations, such as the Department of State Health Services, and the Texas Health and Human Services (HHS) Breast and Cervical Cancer Screening (BCCS) program. Often, clinics use these reports to compare their results with the benchmarks set by the CDC’s Healthy People 2030 goals. Implementing SEMM would mean that clinics would have metrics to show how they were meeting cancer prevention service needs.

### Inner Setting

The inner setting domain includes characteristics of the implementing organization that might influence implementation.

Available resources refer to the level of resources dedicated for implementation and ongoing operations, including money, training, education, physical space, and time [22,23,26,27]. Participants across all levels described examples of available resources (and the lack of these) at their clinic sites. Available resources, described as facilitators, ranged from clinics having established partnerships (see *Cosmopolitanism*) to funding granted by external agencies such as the BCCS program. Importantly, these resources leveraged breast and cervical cancer screening and human papillomavirus (HPV) vaccination efforts at clinic sites. One mid-level participant commented on the ability to reach uninsured patients through BCCS:

We currently have a state-funded grant called breast and cervical cancer screening, (BCCS), that we are able to schedule patients who are uninsured for breast and cervical cancer screening services.

Lack of available resources was described as a barrier, and specifically for SEMM implementation, these included the need for money, limited staff capacity, and space. As one leadership participant put it:

More space is needed for workers. If we had more space, which at both sites right now, we do not. I could not implement something like that [SEMM], because if we’re going to bring on more people, we need space to have them have a place to work.

Compatibility is the degree of tangible fit between the meaning and values attached to the intervention by involved individuals, how those align with individuals’ own norms, values, and perceived risks and needs, and how the intervention fits with existing workflows and systems [23]. Compatibility of SEMM was seen as both a facilitator and barrier. While the SEMM program was perceived as a “good fit” with the clinics’ core mission to promote health education through existing clinic initiatives (eg, well woman exam, mobile mammogram van), it was also viewed as possibly competing with already prioritized specific health topics (eg, diabetes prevention) that clinics were addressing. In light of clinics’ competing priorities, participants were also concerned that SEMM may require resources they lacked (eg, limited staff or team and time), and this was perceived as a potential barrier. One mid-level participant said:

I think that we would need to make sure that we have the right team, the right number of CHWs, or involvement within the program and to make sure that there was enough time allotted to engage in another program. I mean, those are all barriers as far as why the program could be unsuccessful. If we just didn’t have the dedicated time to promote a new program.

Access to knowledge and information. Access to knowledge and information about programs and how to integrate them into work tasks can benefit program implementers [23]. While participants at all levels generally spoke about the importance of accessing knowledge and information (eg, access to external training) and viewed it as a facilitator, one leadership participant suggested that having one dedicated person to introduce the program and facilitate internal communications could be helpful for SEMM implementation. In addition, both mid-level and CHW participants considered ongoing training or education, especially for CHWs, to be important. However, not all clinics provided continuous training for CHWs, and some clinics did not have enough staff or relied on LHWs rather than CHWs to conduct training.

Implementation climate. The capacity of an organization to change and the shared receptivity of the involved individuals are indicative of the implementation climate [23] and a factor in program adoption. Among some leadership and mid-level participants, the implementation climate for new interventions was viewed as a facilitator because their organization’s culture required and supported buy-in from staff and included opportunities for the team to pitch new ideas. Leadership participants spoke about the importance of staff buy-in when implementing new clinic initiatives.

“We just need to make sure that the staff members buy into how they want to do that, and I would really allow them to come up with the way they want to implement it.”

Mid-level participants described having “open door policies” at different levels and having the ability to “bring in new ideas” and share resources.

Networks and communication refer to the nature and quality of webs of social networks and the nature and quality of formal and informal communications within an organization [14]. The nature and the quality of social connections with organizations can influence program implementation [14]. Participants at all levels mentioned the function of networks and communication within their clinical settings and viewed them as beneficial. Leaders described how they generally communicated with their clinic staff and the frequency of those encounters.

We have provider meetings. They meet at least twice a month. The entire staff meets. So, we seek input from the team of people selected to focus on a particular quality measure and then develop something around that.

One CHW shared that giving feedback to her supervisor was required and often helpful,

So every event that I have, I have to complete a form and return it back to my supervisor. We sit down and we talk about it to see how we can work the problem out and solve it, see if there’s anything that we can come up with.

Goals and feedback refer to the degree to which goals are clearly communicated, acted upon, and fed back to staff and alignment of that feedback with goals [26,28,29]. Evaluation and feedback about the clinical and performance outcomes based on program evaluations and patient experience are critical [30]. As such, the degree to which program goals are acted upon, along with evaluation regarding clinical progress, is critical to understanding program implementation [28]. One leader pointed out that the data standards needed to evaluate the quality of intervention uptake may be a potential barrier. This leader suggested that although physicians are expected to report and collect data accurately, some patients don’t fill out self-report questionnaires.

### Characteristics of Individuals

The domain “characteristics about individuals” describes the attitudes, beliefs, and perceptions of people who work within the organization [14]. While some subdomains apply to individuals’ attitudes about the intervention, others describe their perceived self-efficacy to deliver the program and roles within the organization. However, these individual characteristics were not prevalent in the dataset; “other personal attributes” were the only prevalent subdomain within “characteristics about individuals” that was prevalent in the dataset.

Other personal attributes refer to other personal traits such as tolerance of ambiguity, intellectual ability, motivation, values, competence, capacity, innovativeness, tenure, and learning style [14,23]. Interviewees from all levels spoke about how individuals’ attitudes or perspectives were facilitators to the implementation of interventions. Salient individual characteristics included motivation to perform well, or a “heart for service.” One CHW described the attitude necessary for intervention implementation, and public health more broadly:

You have to have it in your heart. You have to have the right spirit in mind. Smile no matter what.

A mid-level staff member also mentioned that it is helpful when leadership staff has a public health background compared to physicians without public health experience.

### Process of Implementation

The process domain describes an organization’s activities and strategies to implement innovations [14]. Particularly, engaging appropriate individuals to champion the implementation of the intervention may improve adoption. Notably, participants frequently discussed people’s roles in program implementation (ie, leaders, stakeholders, and champions).

Formally appointed internal implementation leaders are individuals who are responsible for implementing the intervention in the role of coordinator, project manager, or team leader [14]. A wide range of clinic staff was identified as important implementation leaders, including medical directors, members of the executive team, CEOs, CMOs, clinical directors, billing directors, population health teams, department chairs, chief medical officers, chief nursing officer, HR, chief operating officers, clinical health workers, promotoras, women’s health coordinators, directors, health education directors, and clinic coordinators.

The importance of key stakeholders was another theme that emerged. They were described mostly as facilitators in implementing any new intervention in the clinic. Key stakeholders included customer service associates, clinical staff, CHWs, health education departments, mobile mammogram van programs, health care providers, outreach staff, directors, and other administrators. Key stakeholders were suggested as partners who can be leveraged to facilitate intervention implementation through consultation with clinic leadership before implementing new programs or initiatives in clinical settings. For example, one participant mentioned that, as a leader, she considers the likelihood of staff buy-in before launching any new initiatives (see implementation climate). Participants noted that while stakeholder buy-in can facilitate intervention implementation, not securing buy-in from key stakeholders, such as implementation staff, could potentially result in a major barrier to the implementation of SEMM.

The varying roles of CHWs across clinics also emerged as likely relevant to SEMM implementation. While some clinics have CHWs with clearly specified roles, others had LHWs. CHWs and LHWs were flexible in job function and had a diverse range of roles. CHWs also varied in how much training they had received and in their background knowledge of cancer screening services. Other clinics had no CHWs.

Champions are individuals dedicated to supporting, marketing, and driving through an implementation, overcoming indifference or resistance that the intervention may provoke in an organization [31]. External change agents are individuals who are affiliated with an outside entity who formally influence or facilitate intervention decisions in a desirable direction [14]. Participants also identified both internal champions and external change agents [14] as important to implementation. Relevant champions included quality coordinators, care coordination staff, and clinical health workers. Participants described the need for champions to motivate both patients and staff. For example, when asked what would facilitate SEMM’s implementation at their clinic, one participant described the need for a champion who would “take the bull by the horns and then just drag us with them.” A few participants also noted the role of external change agents in delivering elements of intervention programming, such as university faculty delivering community education and a mobile mammography van providing screenings.

## Discussion

### Principal Findings

Our study presents a unique opportunity to understand the underlying factors relevant to support implementation of an evidence-based cancer screening intervention [32]. Conducting a comprehensive needs and asset assessment, as outlined in Implementation Mapping, is critical for understanding how evidence-based programs, such as SEMM, can be translated effectively across different clinic settings. This qualitative study examined factors relevant to the adoption and implementation of SEMM from the perspective of decision makers and implementers who would consider its uptake and use in primary care clinics. Using the CFIR framework, we identified key barriers and facilitators related to all 5 CFIR domains, which guided the development of SEMM implementation strategies [14]. More specifically, our findings indicate that implementation facilitators fell into the following three CFIR domains: intervention characteristics, outer setting, and inner setting, and implementation barriers fell within the inner setting and intervention characteristics.

Relevant to the outer setting for implementing SEMM as a clinic-based intervention, establishing connections and collaborating with other organizations is critical for supporting outreach programs. Collective networks represent the social capital [33] of an organization to support information-sharing and program implementation. Leveraging these networks can enhance potential resource sharing and help to support new programs [33]. This underscores the importance of understanding how clinics are connected to other service organizations and can be used to select or develop strategies to enhance shared resources, facilitate communication regarding the new program, and support other program needs. The implication is that while clinics operate independently, there is an opportunity to leverage other community partners to support the implementation of new programs such as SEMM.

Another factor relevant to the outer setting is the social context in which the organization exists [14]. In particular, patient-centered organizations are more likely to implement change effectively [34] if they have a deep understanding of how best to meet their patient population’s needs. An integral part of implementing any program that seeks to improve patient outcomes [35] is its emphasis and value on meeting patients’ needs and resources. One approach for meeting patient needs is the employment of CHWs who can address social and cultural barriers to improve health access. Thus, among clinics with CHWs or (eg, any staff member with patient education roles), the role and functions of patient educators within clinics should be known, recognized, and integrated to understand patient needs and optimize program implementation. Given the diverse ways clinics may offer patient education and engage staff as patient educators, SEMM could offer multiple versions of CHW training or tailored training that caters to the varying educational needs of CHWs with different experiences and backgrounds (ie, “beginner training” versus “advanced training”) and is based on the patient needs for education.

Factors relevant to the inner setting also emerged as important facilitators to implementation, particularly related to increasing buy-in from staff across all levels of the organization. Other studies have highlighted the importance of staff [36] as key in decision-making to support implementation efforts as well as leadership involvement [29,37,38]. The sense of team relationships and decision-making may contribute to implementation effectiveness [39]. Thus, buy-in and methods of obtaining buy-in from staff with different roles in the program are critical to the planning process. Additionally, given the diversity of job roles that participants identified as implementation leaders, it may be important to note that formally appointing program “champions” may not be limited to a specific role but would depend on the clinic system team to select the most appropriate leader. Similarly, the Diffusion of Innovation Theory [40] advocates for program champions and those with responsibility for implementation to be included in the planning, as it provides the opportunity for organizations to have a realistic perspective of resources, staffing, and other factors needed for implementation [41].

Characteristics of the individuals involved in implementation are a critical factor. Cooper et al [42] suggested that individual characteristics are an often-overlooked domain within implementation research, and that the role of the individual in impacting the successful implementation of a program may be unrecognized. When thinking about implementation, intervention designers should not assume that all CHWs will have similar training or background knowledge on cancer screening services or that all clinics will have CHWs. Although reliance on individuals’ motivation and a “heart for service” is critical, it could be important for implementation planners to consider what supports are in place to prevent burnout or compassion fatigue. Our results are consistent with other implementation guidelines calling for the assessment of individual characteristics and preferences before intervention delivery [43].

Interviewees highlighted several barriers to implementing SEMM within their clinics. Although the intervention was seen to have a relative advantage, there was a perception of “added work” that came along with any new program. Assessment of current expertise within the organization is critical. While a program may be new to the clinic, the topic and expertise may already be embedded as part of CHW roles. The implication is that program planning teams must work with their clinic staff to assess their clinic capacity, needs, and assets to prepare for implementation of a new program, leveraging their resources, skills, and knowledge to avoid repetition (eg, redundant trainings), and garner support from potential implementers. A needs and assets assessment to prepare for program implementation would also help to address potential implementors’ concerns that, while the new program may be a good fit for the clinic, consideration should be given to ways in which it may compete with programs already in place. Furthermore, our findings indicate that staff perceptions regarding the program’s benefits and rewards play a significant role as facilitators. Understanding how organizations collaborate with others to accomplish their mission may reveal how their networks can be leveraged to support the new program. Additionally, factors like the availability of resources, how compatible the intervention is with the organization’s mission, and the implementation buy-in from others beyond the organization’s leadership are critical. Finally, new programs may require funding and space allocation to be delivered. If not available, it would be highly challenging to implement.

### Limitations

There are a few aspects of the study that limit generalizability. First, this qualitative research included a small sample of participants, representing a small sample of clinics. However, we intentionally included participants within various clinic roles critical to implementation and an adequate number of respondents for thematic saturation [19]. Second, study participants were from clinics based exclusively in urban areas in Texas and may limit generalizability. Third, given that our interviews were conducted in the later part of the COVID-19 pandemic, it is possible that participants’ views regarding taking on a new program may have been influenced. Fourth, given that one clinic previously partnered on SEMM, there is a possibility of self-selection bias. However, all participants shared critical feedback. Finally, our qualitative analysis process did not include member checking. However, in addition to our qualitative approach described above, our Community Advisory Board, comprised of members similar to our participants, reviewed our findings, and we provide direct quotations to accurately reflect participants’ responses.

### Conclusions

Overall, our findings highlight the importance of examining both organizational and individual-level factors in the successful planning process for the implementation of SEMM in clinical settings. Additionally, engaging with different clinic partners helped identify key factors for translating the evidence-based SEMM program, underscoring the importance of engaging with diverse clinic partners to plan implementation. This approach can help align the intervention with the clinic setting and goals, but also secure intervention acceptance, or buy-in. In particular, the degree to which the intervention is adaptable, the need to align the program with the clinic’s goals and capacity to ensure clinic stakeholder buy-in, and the program’s complexity are key considerations for intervention uptake. These findings informed the development and refinement of SEMM implementation support strategies for clinics. The use of the CFIR framework helped to elucidate important factors that will continue to guide implementation strategies.

## Abbreviations

BCCS: breast and cervical cancer screening
CDC: Centers for Disease Control and Prevention
CFIR: Consolidated Framework for Implementation Research
CHW: community health worker
FQHC: federally qualified health center
LHW: lay health worker
SEMM: Salud en Mis Manos
